# Clinical Time Delay Distributions of COVID-19 in 2020–2022 in the Republic of Korea: Inferences from a Nationwide Database Analysis

**DOI:** 10.3390/jcm11123269

**Published:** 2022-06-07

**Authors:** Eunha Shim, Wongyeong Choi, Youngji Song

**Affiliations:** Department of Mathematics, Soongsil University, 369 Sangdo-ro, Donjak-gu, Seoul 06978, Korea; chok10004@soongsil.ac.kr (W.C.); syj7825@soongsil.ac.kr (Y.S.)

**Keywords:** COVID-19, epidemiological distribution, Delta variant, Republic of Korea, serial interval, SARS-CoV-2

## Abstract

Epidemiological distributions of the coronavirus disease 2019 (COVID-19), including the intervals from symptom onset to diagnosis, reporting, or death, are important for developing effective disease-control strategies. COVID-19 case data (from 19 January 2020 to 10 January 2022) from a national database maintained by the Korea Disease Control and Prevention Agency and the Central Disease Control Headquarters were analyzed. A joint Bayesian subnational model with partial pooling was used and yielded probability distribution models of key epidemiological distributions in Korea. Serial intervals from before and during the Delta variant’s predominance were estimated. Although the mean symptom-onset-to-report interval was 3.2 days at the national level, it varied across different regions (2.9–4.0 days). Gamma distribution showed the best fit for the onset-to-death interval (with heterogeneity in age, sex, and comorbidities) and the reporting-to-death interval. Log-normal distribution was optimal for ascertaining the onset-to-diagnosis and onset-to-report intervals. Serial interval (days) was shorter before the Delta variant-induced outbreaks than during the Delta variant’s predominance (4.4 vs. 5.2 days), indicating the higher transmission potential of the Delta variant. The identified heterogeneity in region-, age-, sex-, and period-based distributions of the transmission dynamics of COVID-19 will facilitate the development of effective interventions and disease-control strategies.

## 1. Introduction

Severe acute respiratory syndrome coronavirus-2 (SARS-CoV-2) was first detected in Wuhan City, People’s Republic of China in December 2019 and subsequently spread globally. Until 10 January 2022, SARS-CoV-2 had infected more than 326 million individuals and caused more than 5.5 million deaths [1]. The World Health Organization (WHO) declared coronavirus disease 2019 (COVID-19) a pandemic on 11 March 2020. The SARS-CoV-2 B.1.617.2 (Delta) variant, which was first detected in December 2020, had been dominant globally (since late December 2021) as well as in the Republic of Korea, where the detection rate increased from 1.4% in April 2021 to 90% in August 2021; however, the Omicron sub-lineage BA.1 variant had increasingly replaced the Delta variant by late January 2022 [2].

Estimates in descriptive epidemiology essentially characterize disease spread and thereby enable the implementation of successful containment strategies. Prolonged intervals between infection and eventual case or death reporting obscure the dynamics underlying an outbreak. Understanding such delays is essential for determining the progress of an outbreak in any country. Thus, country-specific estimates of the onset-to-death distribution could help determine the infection fatality ratio and facilitate the prediction of the cumulative mortality during the early stages of a pandemic. Moreover, comparative estimation of serial intervals from before-to-during the predominantly Delta variant-induced outbreaks implicated the high transmissibility and increased prevalence of the Delta variant during the fourth wave of the COVID-19 pandemic in the Republic of Korea.

Initial estimates of COVID-19 epidemiological distributions in the Republic of Korea relied on relatively few data points or limited regional datasets instead of national data [3,4]. National disease surveillance in the past 2 years has yielded more suitable data for re-evaluating the time-delay distributions of COVID-19. Particularly, the availability of individual-level data from a large number of confirmed local cases (*n* = 670,483 by 10 January 2022) in the database maintained by the Korea Disease Control and Prevention Agency (KDCA) and the Central Disease Control Headquarters has facilitated robust statistical estimates of the time-delay distributions of onset-to-death, onset-to-diagnosis, onset-to-report, report-to-death, and serial intervals that enable the calibration of computational models of COVID-19 transmission.

This study was performed to ascertain the epidemiological distributions of the COVID-19 pandemic in the Republic of Korea by quantifying the epidemiological distributions associated with COVID-19. Herein, we aimed to generate an informed, reliable framework for modeling that could guide public health policy. We fitted and analyzed the epidemiological distributions to present the results of fitting at subnational and national levels to a range of probability density functions (PDFs). As our study period includes the Delta-dominant outbreak period during the fourth community-wide epidemic of COVID-19 in the Republic of Korea, we compared the mean serial intervals from before to during the Delta variant’s predominance.

## 2. Materials and Methods

### 2.1. Study Design and Data Analysis

We obtained individual-level records, including information on age, sex, state of residence, presence of underlying health conditions, date of contact with an infector, date of symptom onset and reporting, and date of death, of confirmed COVID-19 cases that were reported to the KDCA and Central Disease Control Headquarters (Table 1 and Table 2). During the study period (19 January 2020 to 10 January 2022), 670,483 confirmed cases were recorded, including 19,418 imported cases and 6071 deaths. During our study period, all COVID-19 cases in Korea were confirmed using the polymerase chain reaction (PCR) tests [5]. The dataset was filtered to obtain the data for onset-to-diagnosis, onset-to-report, onset-to-death, and reporting-to-death. The onset-to-diagnosis data were split into subgroups of cases with and without a known exposure, whereas the onset-to-death data were subclassified into subgroups of cases with and without underlying health conditions. The onset-to-diagnosis and onset-to-report data were aggregated for the seven federal districts of the Republic of Korea (Metro, Chungcheong, Gangwon, Gyeongbuk, Gyeongnam, Honam, and Jeju) for subnational estimates (Table 2).

**Figure 1:**
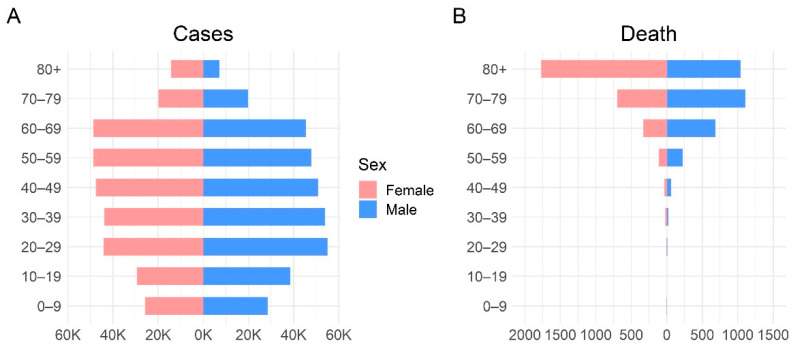
Demographics of patients with COVID-19 in the Republic of Korea. (**A**), number of confirmed COVID-19 cases; (**B**), number of confirmed COVID-19 deaths. COVID-19, coronavirus disease.

Assuming that the entries were incorrectly inputted, we removed rows wherein the date of diagnosis or reporting preceded the onset date. Similarly, entries with a distribution time greater than the study period (723 days) were excluded as they could be typographical errors. After excluding nine individuals with incorrectly inputted entries, we analyzed the onset-to-diagnosis distribution of 405,457 COVID-19 patients. Additional data filtering was applied when the onset-to-death data were analyzed. To exclude cases of deaths caused by diseases other than COVID-19, the entries with onset-to-death distribution times of 0 days or greater than 100 days (12 cases) were removed, and we analyzed the data of cases of 3478 deaths associated with COVID-19. Table 1 summarizes the data, including the number and range of samples, for each variable of interest. The age–sex structure of the confirmed cases is shown in Figure 1. A summary of the number of data samples per region is provided in Table 2.

Number of samples (*N_samples_*) is reported for the whole country.

To estimate the serial intervals, we categorized the study duration into two periods (Period 1: 19 January 2020 to 24 July 2021; Period 2: 25 July 2021 to 10 January 2022), considering that the incidence of the Delta variant among local cases was ≥50% from 25 July 2021. Therefore, we considered two periods to compare the serial intervals from before to during the Delta variant’s predominance. Overall, 189,874 and 480,609 cases were identified in periods 1 and 2, respectively. In these datasets, some case reports included information regarding contact tracing with matched pairs (i.e., case numbers of the infectors and infectees) and dates of symptom onset. The serial interval was defined as the time interval between symptom onset for both the infector and the infectee in the transmission chain. We utilized the *Epicontact R* library to analyze pairwise contact between individuals and estimate the serial interval [6]. Entries with a time interval between symptom onset (for both the infector and the infectee) of fewer than 0 days or greater than 30 days were deleted. We identified 64,578 transmission pairs (30,254 and 34,504 pairs in periods 1 and 2, respectively) that comprised the date of symptom onset for both the infector and infectee, and the transmission pairs were reconstructed according to the date of symptom onset. This study was retrospectively conducted using data obtained for clinical purposes. The Institutional Review Board of Soongsil University waived the need for ethical approval (SSU-202202-HR-381-1).

### 2.2. Model Fitting

Using a nationwide dataset obtained from the KDCA and the Central Disease Control Headquarters, we estimated the following key epidemiological parameters: onset-to-diagnosis, onset-to-report, onset-to-death, reporting-to-death, and serial interval. For onset-to-diagnosis reporting, we performed an additional analysis to separate cases with and without known exposure wherein the index case was infected. Similarly, for the distribution of onset-to-death reporting, we performed an additional analysis to separate cases with and without any underlying health conditions, for males and females and for the cases stratified across six age groups (0–39, 40–49, 50–59, 60–69, 70–79, and ≥80 years).

To determine the best model to fit the data, we adopted a Bayesian model comparison [7,8]. For model selection, the epidemiological distributions were fitted to three probability distributions (gamma, Weibull, and log-normal) that are commonly used to model epidemiological periods [9,10,11]. Specifically, we calculated the Bayes factor $B_{ij}$, which is the likelihood ratio of the marginal likelihood of two competing hypotheses [8]:

$$B_{ij}=\frac{p\left(y|M_{i}\right)}{p\left(y|M_{j}\right)}$$

For readability, we reported Bayes factors as $2log\left(B_{ij}\right)$ according to the notation in the report by Kass and Raftery [8]. Here, $p\left(y|M_{i}\right)$ denotes the evidence of model $M_{i}$, given the data y. To determine the density $p\left(y|M_{i}\right)$, an integral over the model parameters (θ) should be calculated analytically; however, the parameter θ is intractable. Therefore, it should be approximated numerically. Specifically, we approximated $p\left(y|M_{i}\right)$ with $p_{0}\left(y|M_{i}\right)$ where $p_{0}=q\left(\hat{\theta }\right)\sqrt{\det\left(2\pi {\hat{\Sigma }}^{-1}\right)}$ [8,12]. Here, $p_{0}(y|M_{i})$ is based on a second-order Laplace’s method of approximation, $q_{0}(\theta |M_{i},y)$, to the true non-normalized posterior density, $q(\theta |M_{i},y)$:

$$q_{0}=q\left(\hat{\theta }\right)\exp\left(-\frac{1}{2}\left(\theta -\hat{\theta }\right){\hat{\Sigma }}^{-1}{\left(\theta -\hat{\theta }\right)}^{T}\right).$$

Here, $q\left(\hat{\theta }\right)$ is the value of the non-normalized posterior density that is evaluated using the mean estimates of the model’s parameter $\hat{\theta }$, whereas $\hat{\Sigma }$ is the covariance matrix built from the Markov Chain Monte Carlo (MCMC) samples of the posterior distribution.

We employed a hierarchical Bayesian model with partial pooling to estimate the model parameters at the region level. The parameters of the considered distributions at the national level were estimated by fitting each PDF to the fully pooled data using data for all regions. The prior probabilities for the national-level parameters for each considered PDF were chosen to be $N^{+}\left(0,1\right)$, where $N^{+}\left(\cdot \right)$ was a truncated normal distribution. Subsequently, we estimated the model parameters at the regional level as a sample from a distribution of national-level parameters. Considering the gamma distribution as an example, the gamma distribution for the ith region is denoted as $Gamma\left(\alpha_{i},\beta_{i}\right)$. Parameters $\alpha_{i}$ and $\beta_{i}$ were assumed to be normally distributed random variables as follows:

$$\alpha_{i}\sim N\left(\alpha_{Korea},\sigma_{1}\right)$$

$$\beta_{i}\sim N\left(\beta_{Korea},\sigma_{2}\right)\;$$

where $\alpha_{Korea}$ and $\beta_{Korea}$ are parameters for the national level and $\sigma_{1}\sim N^{+}\left(0,1\right)$ and $\sigma_{2}\sim N^{+}\left(0,1\right)$ are assumed. Additionally, for all the fitted densities, the mean and variance parameters were constrained to be positive. Posterior samples of parameters for each distribution were obtained using the Hamiltonian Monte Carlo method with Stan [13,14]. For each fit, four chains were used with 2000 iterations, half of which were dedicated to the warm-up. Data cleaning was conducted using R (v. 4.0.5, R Foundation for Statistical Computing, Vienna, Austria) [15], and the distribution was estimated using Python (v. 3.8.8, Python Software Foundation, Wilmington, DE, USA) [16]. The PyStan (v. 2.19.0.0) [17] interface was employed to fit the model with Stan [18,19].

## 3. Results

### 3.1. Estimation of National Epidemiological Periods

**Figure 2:**
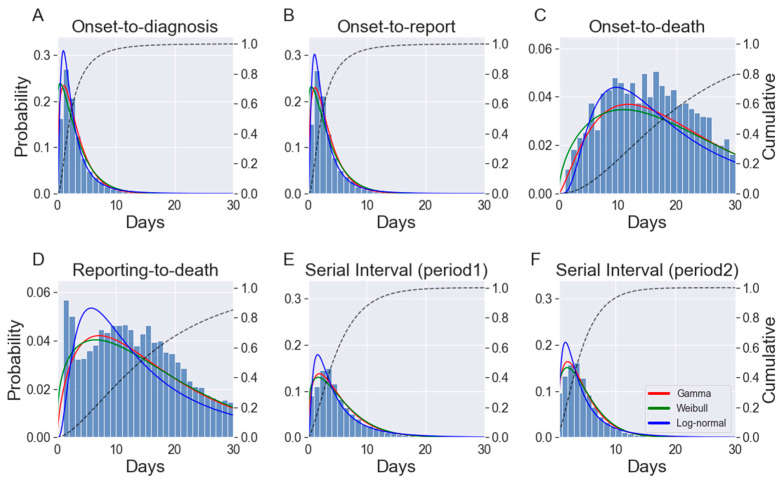
Histograms and the distribution of: (**A**), onset-to-diagnosis; (**B**), onset-to-report; (**C**), onset-to-death; (**D**), reporting-to-death; (**E**), serial intervals in Period 1; and (**F**), serial intervals in Period 2. Note: Solid lines indicate fitted PDFs; dashed lines show the cumulative distribution function of the best-fitting PDF. The *y*-axis on the left-hand side shows the probability value of the PDFs, and the *y*-axis on the right-hand side shows the value of the cumulative distribution function. All values on the *x*-axis are presented in days. PDF, probability density functions.

The time from symptom onset to diagnosis and report were recorded for 405,457 and 406,355 cases, respectively, out of the 670,483 cases that occurred nationwide (Table 1). During the study period, 6071 COVID-19-related deaths were reported in the Republic of Korea, and the time from symptom onset to report and death were available for 3478 and 5941 cases, respectively (Table 1). Three PDFs (gamma, Weibull, and log-normal) were fitted to the epidemiological periods and serial intervals (Table 3 and Figure 2).

The model fits were tested using the Bayes factor, which was employed for model selection. The log-normal PDF provided the best fit for onset-to-diagnosis (all cases, cases with known exposure, and cases without known exposure) and onset-to-report. However, for the distributions of reporting-to-death, serial interval, and onset-to-death in all fatal cases, cases with underlying diseases, and cases without underlying diseases, the gamma distribution was the preferred model. The results of the fitted probability distributions with the corresponding parameter estimates (mean, variance, PDF parameter values, and 95% credible intervals [CrI]) are listed in Table 3.

For each epidemiological distribution that was considered, the cumulative probability distribution was provided for the best-fit model, which revealed that 90% of cases were diagnosed and reported within 7 days of symptom onset (Figure 2). Moreover, 84% and 91% of the serial intervals in periods 1 and 2, respectively, were shorter than 9 days. Furthermore, of the terminally ill patients with COVID-19, approximately 80% died within 30 days of symptom onset. We estimated a case fatality ratio (CFR) of 0.91%, which is lower than the global average reported by the WHO (1.78%) [1]. The CFR in males and females were similar (0.90% and 0.92%, respectively) [20]. The age-specific CFR ranged from 0.01% for individuals aged ≤19 years to 14.13% for individuals aged ≥80 years [20].

The mean onset-to-diagnosis time was 3.12 (95% CrI: 3.12–3.14) days for all reported cases; however, it was shorter (2.96 days, 95% CrI: 2.95–2.98 days) for cases with a known exposure (Table 3 and Figure S1 in Supplementary Material). For cases without a known source of exposure, the mean (95% CrI) onset-to-diagnosis time was 3.31 (3.30–3.32) days (Table 3 and Figure S1), and the mean (95% CrI) onset-to-report time was 3.18 (3.18–3.19) days. Analysis of 3478 fatal cases yielded a gamma likelihood with a mean (95% CrI) of 20.57 (20.14–21.04) days and a shape parameter (95% CrI) of 2.35 (2.24–2.45) days that could adequately fit the data. Moreover, the mean onset-to-death time (20.19 days) was shorter for patients with underlying diseases than for those without underlying diseases (22.51 days). The mean (95% CrI) reporting-to-death time was 17.20 (16.86–17.55) days.

### 3.2. Subnational Estimation of Epidemiological Periods

**Figure 3:**
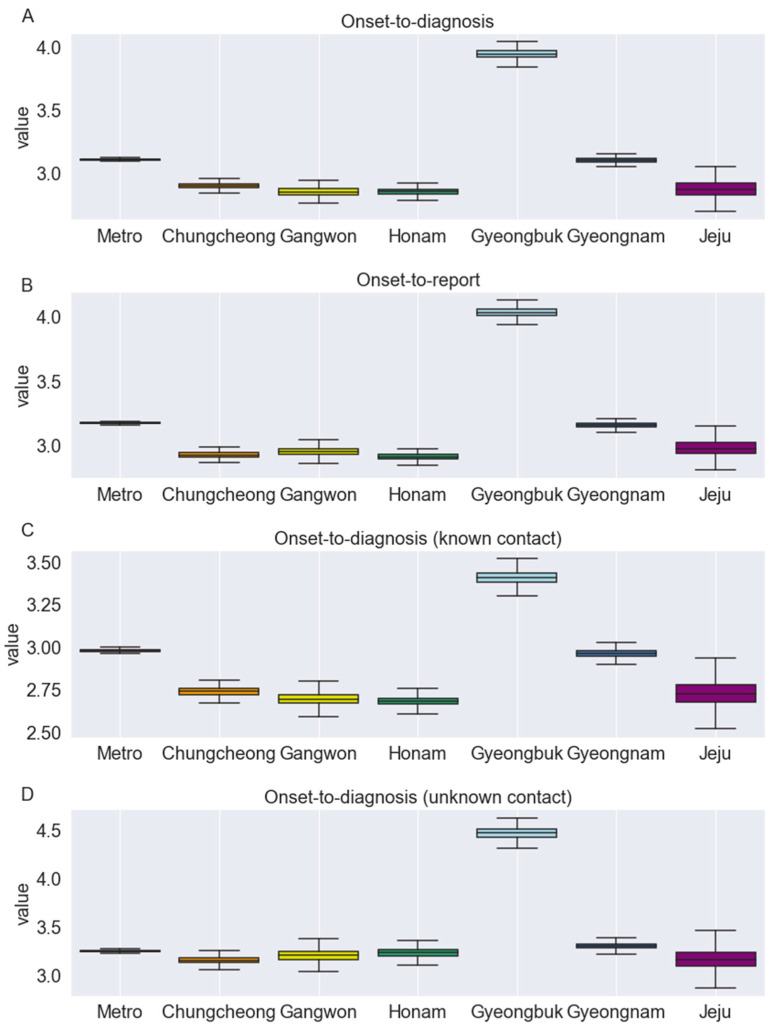
Region-specific estimates of the mean interval (in days) for: (**A**), onset-to-diagnosis (all cases considered); (**B**), onset-to-diagnosis (for cases with known contact); (**C**), onset-to-diagnosis (for cases with unknown contact); and (**D**), onset-to-report distributions fitted in the joint model for the Republic of Korea.

Using partial pooling, the onset-to-diagnosis and onset-to-report distributions were fitted in a joint model across seven federal districts (Metro, Chungcheong, Gangwon, Gyeongbuk, Gyeongnam, Honam, and Jeju) of the Republic of Korea (Table 4). Figure 3 illustrates the subnational variability in the distributions of these epidemiological periods. For example, Honam had the shortest mean onset-to-diagnosis (all cases, regardless of the knowledge of exposure) and onset-to-report time, whereas Gyeongbuk had the longest mean estimates for both aforementioned parameters. Specifically, the mean (95% CrI) onset-to-report time ranged from 2.91 (2.86–2.96) days for Honam to 4.04 (3.97–4.11) days for Gyeongbuk. Additionally, the mean onset-to-diagnosis (all cases) ranged from 2.86 days for Honam and Gangwon combined to 3.95 days for Gyeongbuk (Table 4). For all considered distributions, Metro had the closest mean estimates to the national mean. Furthermore, for all of the considered regions, the mean onset-to-diagnosis time was shorter in cases with a known source of exposure than in those without a known source of exposure.

### 3.3. Age- and Sex-Stratified Time from Onset to Death

The distribution, by sex and age, of the onset-to-death time was determined (Table 3). During the study period, the date of symptom onset was known for 3478 cases of the 6071 fatal cases. Comparison of the mean interval from symptom onset to death for men and women using Welch’s 2-sample *t*-test revealed a significantly longer interval for men than for women (*p* = 5.06 × 10^−10^).

Regarding age-related variations, the mean interval from onset to death peaked among individuals in their 50s and steadily decreased inversely with increasing age (Table 3) in the analysis of the following age groups: 0–39, 40–49, 50–59, 60–69, 70–79, and ≥80 years. The first age group included all fatal COVID-19 cases in patients younger than 40 years. Because of the smaller number of fatal cases, the first age group, which comprised the youngest participants, had a wider age interval than the other age groups and allowed a meaningful evaluation of epidemiological characteristics. The mean time from onset to death could be accurately modeled by a gamma distribution, which estimated the shortest time for the age group ≥80 years at 17.76 days (95% CrI: 17.22–18.31; Table 3).

### 3.4. Serial Interval

The serial interval is a crucial epidemiological variable that characterizes the spread of infectious diseases. The mean and median serial intervals of the total study population were 4.31 (SD, 3.94) and 3 days, respectively. Furthermore, we estimated the serial interval distribution during Periods 1 and 2 by fitting the gamma distribution, which was favorable for serial interval (Table 3). The mean serial interval was shorter in Period 2 (4.43 days) than in Period 1 (5.24 days), indicating that the Delta variant has a growth advantage over the original virus strain, which leads to a faster succession of infection. The estimates of the mean serial intervals were longer in Periods 1 and 2 than in the early stages of the outbreak in the Republic of Korea (4.0 days) [21,22].

## 4. Discussion

This study demonstrated that the COVID-19 pandemic in the Republic of Korea was characterized by relatively short onset-to-diagnosis and onset-to-report intervals but by long serial intervals. A comprehensive understanding of the clinical time delays associated with SARS-CoV-2 infections can facilitate policy decisions for containment and suppressing transmission. We used the most complete individual-level data that are presently available together with Bayesian hierarchical models to estimate various key epidemiological distributions of the COVID-19 epidemic in the Republic of Korea. We fitted three PDFs to the data from the national database to determine several epidemiological variables, including onset-to-diagnosis and onset-to-death, in the Republic of Korea. The study results are consistent with those of a previous study [4] and indicate that the log-normal is preferable for onset-to-diagnosis and onset-to-report intervals. However, the gamma distribution best captured the distribution of onset-to-death and serial intervals. The results discussed above facilitated a clearer understanding of the pandemic in the Republic of Korea and other high-income countries. Furthermore, reporting delays and the shape of the epidemic curve can be incorporated into forecasting models such as the R package Nowcasting by Bayesian Smoothing (NobBS) [23]. Specifically, the correction for underestimation of cases caused by delays in reporting and the estimation of the distribution of delays can potentially improve the accuracy of forecasting models.

Compared to the findings in other countries [18,24,25], this study revealed relatively short onset-to-diagnosis and onset-to-report as well as long serial intervals, which highlights the effectiveness of the reporting system and interventional strategies in the Republic of Korea. The reported mean incubation period of COVID-19 ranges from 4.8 to 9.0 days [weighted pooled mean 6.5 days (95% CrI: 5.9–7.1 days)]; however, the estimated serial interval for both periods in our study was shorter than the mean incubation period [26], which indicates that pre-symptomatic transmission frequently occurred in the Republic of Korea. This result is consistent with the finding of a previous study, which revealed that pre-symptomatic transmission contributed to 37% of all SARS-CoV-2 transmission in the country [27]. Furthermore, the estimated mean serial interval in Period 2 (4.43 days), when the Delta variant was dominant, was shorter than that in Period 1 (5.24 days), implying more rapid and intense transmission of the Delta variant [28]; this agrees with the results of other studies, revealing a shorter serial interval for the Delta variant than for the wild-type virus in the Republic of Korea [4,21,22].

In this study, we observed differences in the mean interval from symptom onset to death between age groups. Furthermore, the 50–59 age group has the longest time from symptom onset to death, with a mean of 25.49 days (95% CrI: 23.14–28.04), while the 80–89 age group had the shortest period at 17.76 days (95% CrI: 17.22–18.31). The distribution of the time delays from symptom onset to death demonstrates that individuals in 40–49 age band have relatively short time delays, which reveals the predominantly more vulnerable nature of adults in this age band, who require either clinical intervention or develop a severe reaction to SARS-CoV-2 infection that results in mortality [29].

Our study had certain limitations. Firstly, we did not consider the effect of COVID-19 vaccinations, although approximately 84% and 41% of the population of the Republic of Korea is fully vaccinated and received boosters, respectively, until 10 January 2022 [20]. Until early January 2022, no vaccination program had been implemented for individuals younger than 12 years. Secondly, changes in social distancing measures that were implemented in the Republic of Korea [20] and varied over time were not considered; enhanced social distancing, including the limiting of gatherings to four persons, was implemented nationwide from 18 December 2021 to 16 January 2022 [20]. Thirdly, reinfection may affect time delay as it is more likely to result in mild symptoms or asymptomatic infections than initial infection [30]. However, reinfection was relatively rarely reported in Republic of Korea, with 8 and 545 cases reported before and during the predominance of the Delta variant, respectively; therefore, it was not analyzed separately [31]. Hence, further study incorporating the changes in the social distancing protocols and vaccination coverage levels could enhance the understanding of the epidemiological characteristics of COVID-19.

## 5. Conclusions

We used the most extensive nationwide COVID-19 database and fitted epidemiological distributions in a hierarchical Bayesian model with partial pooling to estimate the epidemiological distributions of the COVID-19 pandemic in the Republic of Korea. Relatively short onset-to-diagnosis and onset-to-report intervals, as well as long serial intervals, confirmed the effectiveness of the reporting system and interventional strategies. Moreover, heterogeneity in the distributions by region, age, sex, and study period was noted and identified by comparing the parametric forms employed to fit each epidemiological distribution. Accordingly, identifying the time delay for COVID-19 onset and death data provides an informed and reliable framework for modeling and public health policymaking.

## Figures and Tables

**Table 1:** Summary of the distribution data analyzed in the study.

Distribution	*N_samples_*	Range (Days)	Mean (Days)
Onset-to-diagnosis	405,457	0–447	2.6
With known exposure	221,604	0–398	2.43
Without known exposure	183,853	0–447	2.8
Onset-to-report	406,355	0–447	2.66
Onset-to-death	3478	1–97	20.07
With underlying conditions	2912	1–97	19.69
Without underlying conditions	566	1–97	22.02
Onset-to-death (age, in years)	3478	1–97	20.07
0–39	29	1–65	20.79
40–49	44	2–57	19.5
50–59	172	1–81	24.97
60–69	634	1–97	22.82
70–79	1044	1–92	21.78
≥80	1555	1–97	17.27
Onset-to-death (sex)	3478	1–97	20.07
Male	1877	1–97	21.4
Female	1601	1–93	18.51
Reporting-to-death	5941	1–99	16.71
Serial interval			
Period 1	30,254	0–30	4.74
Period 2	34,504	0–30	3.93

Number of samples (*N*_*samples*_) is reported for the whole country.

**Table 2:** Number of datapoints per region for each analyzed dataset.

Regions	Population Density (per km^2^)	Onset-to-Diagnosis	Onset-to-Diagnosis (Cases with Known Exposure)	Onset-to-Diagnosis (Cases without Known Exposure)	Onset-to-Report
Metro	679	316,337	168,605	147,732	316,984
Chungcheong	104	18,759	11,449	7310	18,796
Gangwon	90	7876	5441	2435	7905
Gyeongbuk	1306	18,714	9060	9654	18,799
Gyeongnam	156	27,906	16,371	11,535	27,965
Honam	50	13,735	9323	4412	13,764
Jeju	362	2130	1355	775	2142

**Table 3:** Preferred PDFs with the largest Bayesian support for each COVID-19 distribution, with the estimated mean, variance, and other parameters of the PDF.

Distribution	Model	Mean (95% CrI), Days	Variance (95% CrI), Days^2^	*p*_1_ (95% CrI)	*p*_2_ (95% CrI)
Onset-to-diagnosis					
All	Log-normal	3.12 (3.12–3.14)	10.36 (10.25–10.47)	0.78 (0.78–0.78)	0.85 (0.85–0.85)
Cases with known exposure		2.96 (2.95–2.98)	9.94 (9.79–10.09)	0.71 (0.70–0.71)	0.87 (0.87–0.87)
Cases without known exposure		3.31 (3.30–3.32)	10.43 (10.27–10.59)	0.86 (0.86–0.87)	0.82 (0.81–0.82)
Onset-to-report	Log-normal	3.18 (3.18–3.19)	10.33 (10.22–10.44)	0.81 (0.80–0.81)	0.84 (0.84–0.84)
Onset-to-death					
All	Gamma	20.57 (20.14–21.04)	180.62 (169.43–192.64)	2.35 (2.24–2.45)	0.11 (0.11–0.12)
Cases with underlying conditions		20.19 (19.72–20.68)	173.17 (161.96–185.27)	2.36 (2.24–2.47)	0.12 (0.11–0.12)
Cases without underlying conditions		22.51 (21.31–23.74)	216.52 (185.36–252.51)	2.35 (2.10–2.61)	0.10 (0.09–0.12)
Onset-to-death (age, in years)					
0–39	Gamma	21.31 (16.21–27.90)	264.74 (132.13–531.39)	1.84 (1.13–2.66)	0.09 (0.05–0.13)
40–49		20.00 (15.70–25.33)	265.38 (143.97–474.08)	1.59 (1.05–2.19)	0.08 (0.05–0.12)
50–59		25.49 (23.14–28.04)	270.64 (207.52–351.73)	2.43 (2.01–2.91)	0.10 (0.08–0.12)
60–69		23.32 (22.16–24.51)	241.67 (208.88–277.86)	2.26 (2.04–2.49)	0.10 (0.09–0.11)
70–79		22.29 (21.45–23.18)	204.14 (183.02–228.36)	2.44 (2.26–2.63)	0.11 (0.10–0.12)
≥80		17.76 (17.22–18.31)	125.28 (114.22–137.60)	2.52 (2.36–2.70)	0.14 (0.13–0.15)
Onset-to-death (sex)					
Male	Gamma	21.91 (21.24–22.57)	209.36 (192.00–228.25)	2.30 (2.16–2.43)	0.10 (0.10–0.11)
Female		19.01 (18.45–19.60)	146.88 (134.41–160.50)	2.46 (2.31–2.63)	0.13 (0.12–0.14)
Reporting-to-death	Gamma	17.20 (16.86–17.55)	172.86 (164.32–182.27)	1.71 (1.66–1.77)	0.10 (0.10–0.10)
Serial interval					
Period 1	Gamma	5.24 (5.20–5.29)	17.30 (16.91–17.70)	1.59 (1.57–1.61)	0.30 (0.30–0.31)
Period 2		4.43 (4.39–4.47)	10.88 (10.67–11.11)	1.80 (1.78–1.83)	0.41 (0.40–0.41)

For mean and variance, the 95% credible intervals (CrI) are presented in parentheses. The parameters *p*_1_ and *p*_2_ for the preferred PDFs gamma and log-normal are expressed in the form $gamma\left(x|p_{1},\;p_{2}\right)=gamma\left(\alpha ,\;\beta \right)$ and $lognormal\left(x|p_{1},\;p_{2}\right)=lognormal\left(\mu ,\;\sigma \right)$, respectively, with the formula of the PDFs given in Table S1. PDF, probability density functions.

**Table 4:** Regional-level estimates for the best-fit distributions.

Distribution	Region	Mean (95% CrI), Days	Variance (95% CrI), Days^2^	*p*_1_ (95% CrI)	*p*_2_ (95% CrI)
Onset-to-diagnosis	Metro	3.11 (3.10–3.13)	9.70 (9.59–9.81)	0.79 (0.79–0.79)	0.83 (0.83–0.83)
	Chungcheong	2.91 (2.86–2.95)	9.73 (9.24–10.24)	0.68 (0.67–0.70)	0.88 (0.87–0.88)
	Gangwon	2.86 (2.79–2.93)	9.99 (9.22–10.82)	0.65 (0.63–0.67)	0.89 (0.88–0.91)
	Gyeongbuk	3.95 (3.88–4.02)	27.52 (25.92–29.19)	0.86 (0.85–0.88)	1.01 (1.00–1.02)
	Gyeongnam	3.11 (3.07–3.15)	11.40 (10.92–11.89)	0.74 (0.73–0.75)	0.88 (0.88–0.89)
	Honam	2.86 (2.81–2.91)	8.73 (8.22–9.24)	0.69 (0.67–0.70)	0.85 (0.84–0.86)
	Jeju	2.88 (2.75–3.01)	9.98 (8.55–11.62)	0.66 (0.63–0.70)	0.89 (0.86–0.91)
	Korea	3.13 (3.12–3.13)	10.36 (10.25–10.47)	0.78 (0.78–0.78)	0.85 (0.85–0.85)
Onset-to-diagnosis (cases with known exposure)	Metro	2.98 (2.97–2.99)	9.73 (9.57–9.90)	0.72 (0.72–0.73)	0.86 (0.86–0.86)
	Chungcheong	2.74 (2.69–2.79)	8.91 (8.34–9.52)	0.62 (0.60–0.63)	0.88 (0.87–0.90)
	Gangwon	2.70 (2.62–2.78)	9.13 (8.30–10.06)	0.59 (0.56–0.61)	0.90 (0.89–0.92)
	Gyeongbuk	3.41 (3.33–3.49)	17.40 (16.03–18.85)	0.77 (0.75–0.79)	0.96 (0.94–0.97)
	Gyeongnam	2.96 (2.92–3.01)	10.85 (10.27–11.48)	0.68 (0.67–0.70)	0.90 (0.89–0.91)
	Honam	2.68 (2.63–2.74)	7.61 (7.11–8.18)	0.63 (0.61–0.64)	0.85 (0.84–0.86)
	Jeju	2.73 (2.58–2.89)	9.30 (7.68–11.23)	0.60 (0.55–0.65)	0.90 (0.87–0.93)
	Korea	2.96 (2.95–2.98)	9.94 (9.79–10.08)	0.71 (0.70–0.71)	0.87 (0.87–0.87)
Onset-to-diagnosis (cases without known exposure)	Metro	3.26 (3.24–3.27)	9.30 (9.14–9.45)	0.87 (0.86–0.87)	0.79 (0.79–0.80)
	Chungcheong	3.16 (3.09–3.23)	10.65 (9.84–11.56)	0.79 (0.77–0.81)	0.85 (0.84–0.87)
	Gangwon	3.21 (3.09–3.34)	11.21 (9.74–12.85)	0.80 (0.77–0.83)	0.86 (0.83–0.88)
	Gyeongbuk	4.47 (4.36–4.59)	39.84 (36.51–43.63)	0.95 (0.93–0.97)	1.05 (1.03–1.06)
	Gyeongnam	3.31 (3.25–3.37)	11.74 (11.03–12.49)	0.83 (0.82–0.85)	0.85 (0.84–0.86)
	Honam	3.24 (3.15–3.34)	10.96 (9.90–12.13)	0.82 (0.79–0.84)	0.85 (0.83–0.86)
	Jeju	3.17 (2.97–3.39)	10.78 (8.46–13.63)	0.79 (0.74–0.85)	0.85 (0.81–0.90)
	Korea	3.31 (3.30–3.33)	10.43 (10.27–10.59)	0.86 (0.86–0.87)	0.82 (0.82–0.82)
Onset-to-report	Metro	3.17 (3.16–3.18)	9.67 (9.56–9.78)	0.82 (0.82–0.82)	0.82 (0.82–0.82)
	Chungcheong	2.93 (2.89–2.97)	9.72 (9.24–10.22)	0.70 (0.68–0.71)	0.87 (0.86–0.88)
	Gangwon	2.95 (2.89–3.02)	10.11 (9.36–10.93)	0.70 (0.68–0.72)	0.88 (0.86–0.89)
	Gyeongbuk	4.04 (3.97–4.11)	27.24 (25.67–28.89)	0.90 (0.89–0.92)	0.99 (0.98–1.00)
	Gyeongnam	3.16 (3.12–3.19)	11.33 (10.85–11.81)	0.77 (0.76–0.78)	0.87 (0.86–0.88)
	Honam	2.91 (2.86–2.96)	8.76 (8.27–9.27)	0.71 (0.70–0.73)	0.84 (0.83–0.85)
	Jeju	2.98 (2.86–3.11)	10.52 (9.08–12.20)	0.70 (0.67–0.74)	0.88 (0.86–0.91)
	Korea	3.18 (3.17–3.19)	10.33 (10.22–10.44)	0.81 (0.80–0.81)	0.84 (0.84–0.84)

Mean, variance, and parameter values with 95% credible intervals (CrI). The parameters *p*_1_ and *p*_2_ for the preferred PDFs log-normal are expressed in the form $lognormal\left(x|p_{1},\;p_{2}\right)=lognormal\left(\mu ,\;\sigma \right)$.

## Data Availability

Not applicable.

## Supplementary Materials

The following supporting information can be downloaded at: https://www.mdpi.com/article/10.3390/jcm11123269/s1, Figure S1: Histograms and the distribution of: (A), onset-to-diagnosis (for cases with known contact); (B), onset-to-diagnosis (for cases without known contact); (C), onset-to-death (for cases with underlying conditions); and (D), onset-to-death (for cases without underlying conditions); Figure S2. Cumulative histograms and the cumulative distribution of: (A), onset-to-diagnosis; (B), onset-to-report; (C), onset-to-death; (D), reporting-to-death; (E), serial intervals in Period 1; and (F), serial intervals in Period 2; Table S1: probability density functions with the analytical formula for mean and variance.
