# Supplementary material for: Clinical Time Delay Distributions of COVID-19 in 2020–2022 in the Republic of Korea: Inferences from a Nationwide Database Analysis
Source: J Clin Med. 2022 Jun 7;11(12):3269. doi: 10.3390/jcm11123269 (PMC9225637; doi:10.3390/jcm11123269)
Supplement: Supplementary file 1 [file jcm-11-03269-s001.zip › jcm-1714967-supplementary.pdf]

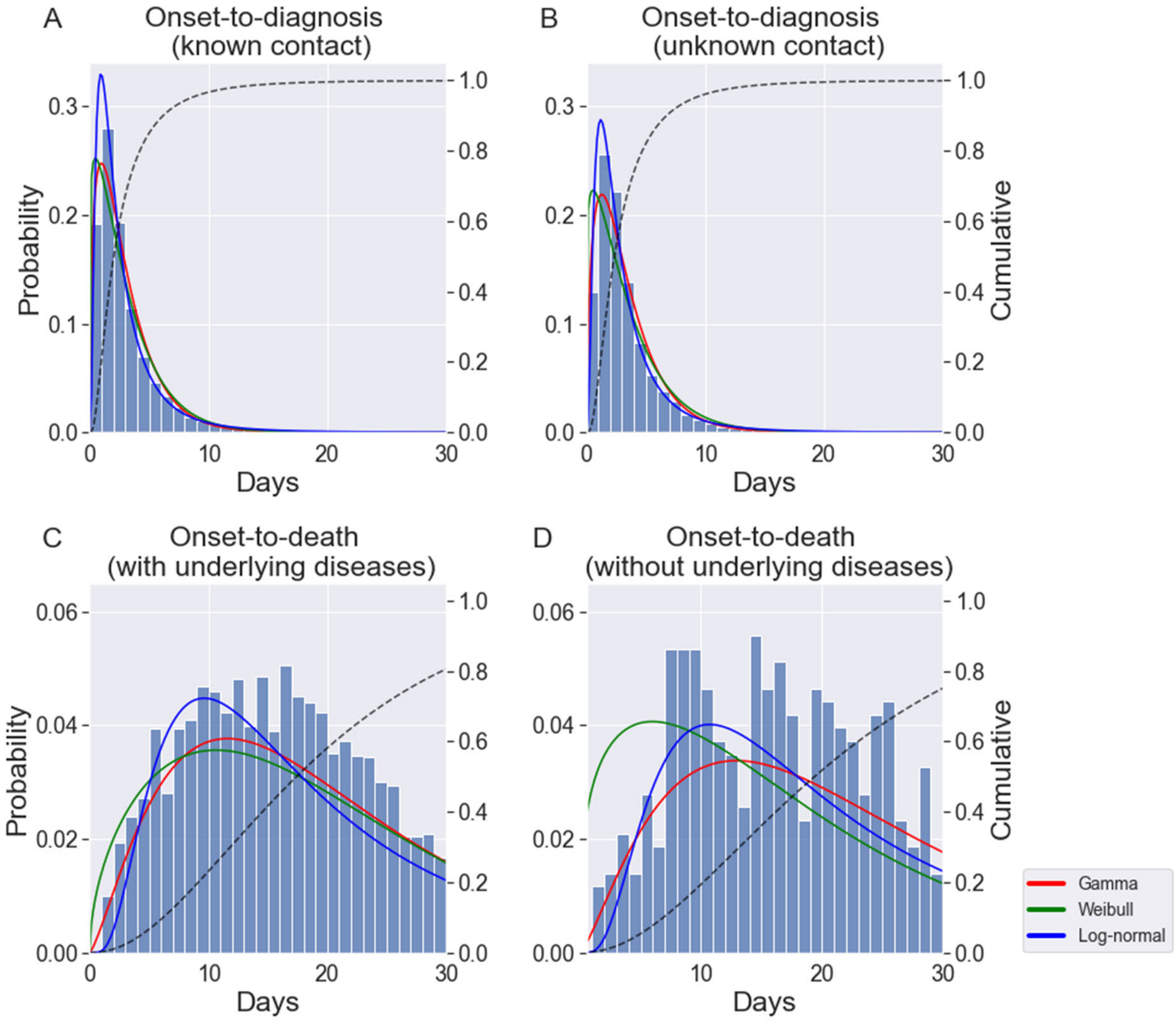

Figure S1. Histograms and the distribution of: **A,** onset-to-diagnosis (for cases with known contact); **B,** onset-to-diagnosis (for cases without known contact); **C,** onset-to-death (for cases with underlying conditions); and **D,** onset-to-death (for cases without underlying conditions). Note: Solid lines indicate fitted PDFs; dashed lines show the cumulative distribution function of the best-fitting PDF. The left-hand side y-axis shows the probability value of the PDFs and the right-hand side y-axis shows the value of the cumulative distribution function. All values on the x-axis are in days.

* PDF, probability density function

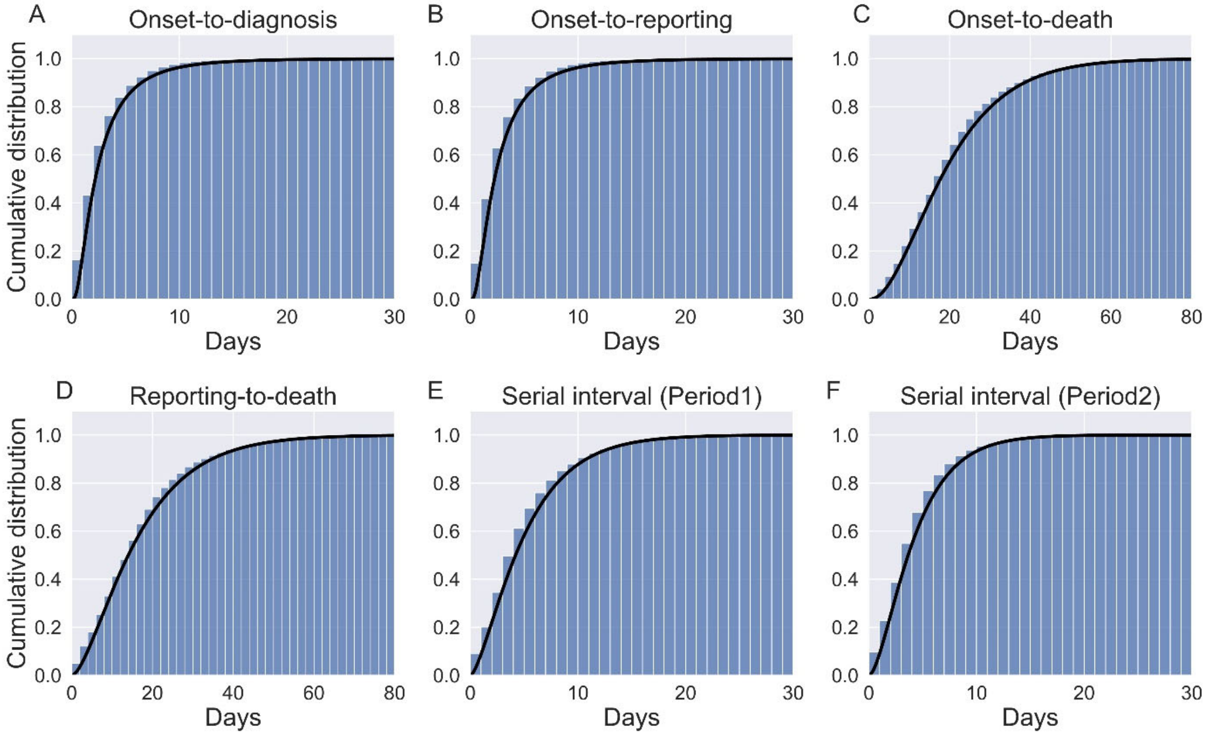

Figure S2. Cumulative histograms and the cumulative distribution of: **A**, Onset-to-diagnosis; **B**, Onset-to-reporting; **C**, Onset-to-death; **D**, Reporting-to-death; **E**, Serial intervals in Period 1; and **F**, Serial intervals in Period 2. Note: Solid lines indicate fitted CDFs; All values on the x-axis are in days.

* CDF, cumulative density function

Table S1. Probability density functions with analytical formula for mean and variance. x denotes the data, $\Gamma(\cdot)$ is a gamma function.

PDF	Mean	Variance
$gamma(x\|\alpha,\beta) = x^{\alpha-1}\frac{\beta^{\alpha} exp\,(-\beta x)}{\Gamma(\alpha)}$	$\frac{\alpha}{\beta}$	$\frac{\alpha}{\beta^2}$
$Weibull(x\|\alpha,\sigma) = \frac{\alpha}{\sigma}\left(\frac{x}{\sigma}\right)^{\alpha-1} exp\left(-\left(\frac{x}{\sigma}\right)^{\alpha}\right)$	$\sigma\Gamma\left(1+\frac{1}{\alpha}\right)$	$\sigma^2\left(\Gamma\left(1+\frac{2}{\alpha}\right)-\left(\Gamma\left(1+\frac{1}{\alpha}\right)\right)^2\right)$
$lognormal(x\|\mu,\sigma) = \frac{1}{x\sigma\sqrt{2\pi}} exp\left(-\frac{(logx-\mu)^2}{2\sigma^2}\right)$	$exp\left(\mu+\frac{\sigma^2}{2}\right)$	$\left(exp\left(\sigma^2\right)-1\right) exp\left(2\mu+\sigma^2\right)$
